# Supplementary material for: High-throughput functional evaluation of BRCA2 variants of unknown significance
Source: Nat Commun. 2020 May 22;11:2573. doi: 10.1038/s41467-020-16141-8 (PMC7244490; doi:10.1038/s41467-020-16141-8)
Supplement: Supplementary file 3 — Description of Additional Supplementary Files [file 41467_2020_16141_MOESM3_ESM.pdf]

Title: Supplementary Data 1:

Description: Information of 107 initial variants and empty vector.

Title: Supplementary Data 2:

Description: Reclassifications of IARC Class 1/2/4/5 BRCA2 variants by ClinVar, ACMG, and MANO-B method.

Title: Supplementary Data 3:

Description: Information of 244 variants and the empty vector.

Title: Supplementary Data 4:

Description: Raw data of 7,344 relative viability values produced by MANO-B method.

Title: Supplementary Data 5:

Description: Functional classification by MANO-B methods with four drugs.

Title: Supplementary Software:

Description: Source codes and raw data for Bayesian inference.
