# Supplementary figures and images for: High-throughput functional evaluation of BRCA2 variants of unknown significance
Source: Nat Commun. 2020 May 22;11:2573. doi: 10.1038/s41467-020-16141-8 (PMC7244490; doi:10.1038/s41467-020-16141-8)

## ROC curve with niraparib

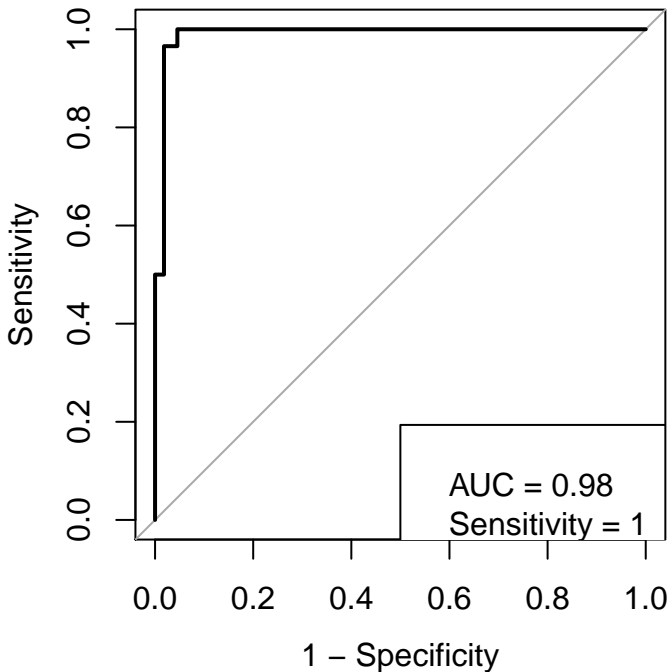

Supplement: Supplementary file 9 — Supplementary Software 1 [file 41467_2020_16141_MOESM9_ESM.zip › compute_code/output/Supp_Fig_5/ROC_nira.pdf]

## ROC curve with CBDCA

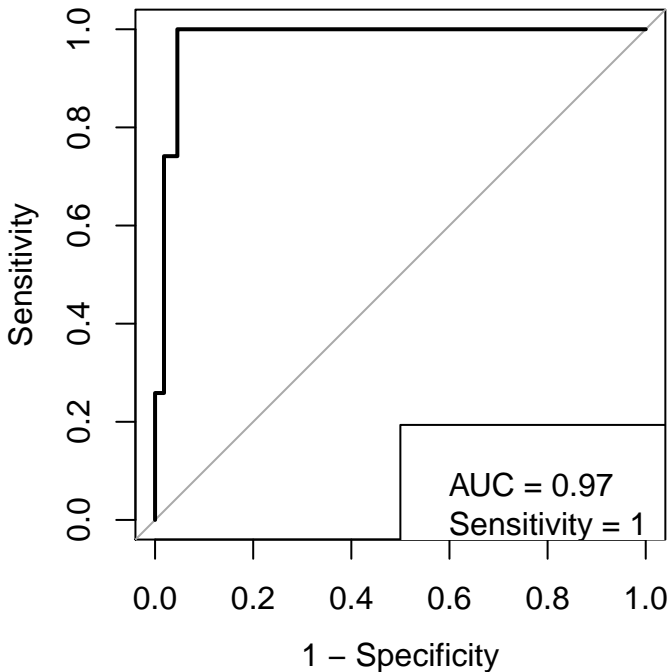

Supplement: Supplementary file 9 — Supplementary Software 1 [file 41467_2020_16141_MOESM9_ESM.zip › compute_code/output/Supp_Fig_5/ROC_CBDCA.pdf]

**ROC curve with rucaparib**

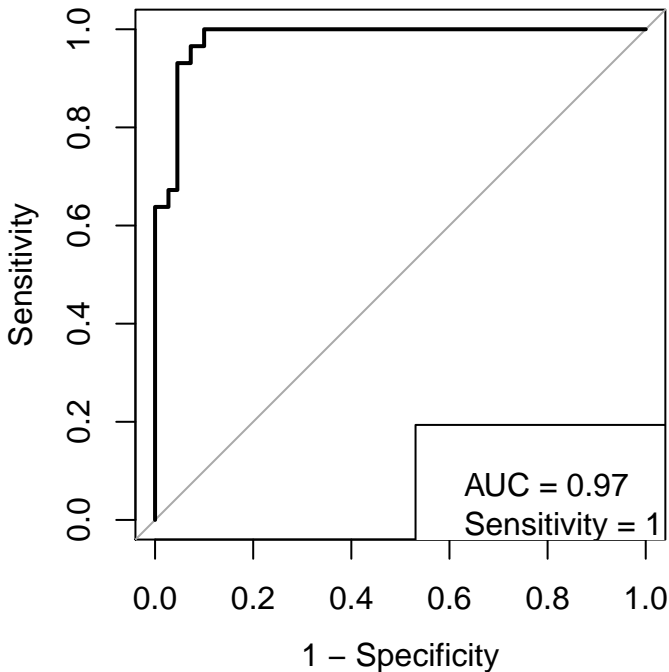

Supplement: Supplementary file 9 — Supplementary Software 1 [file 41467_2020_16141_MOESM9_ESM.zip › compute_code/output/Supp_Fig_5/ROC_ruca.pdf]

**ROC curve with olaparib**

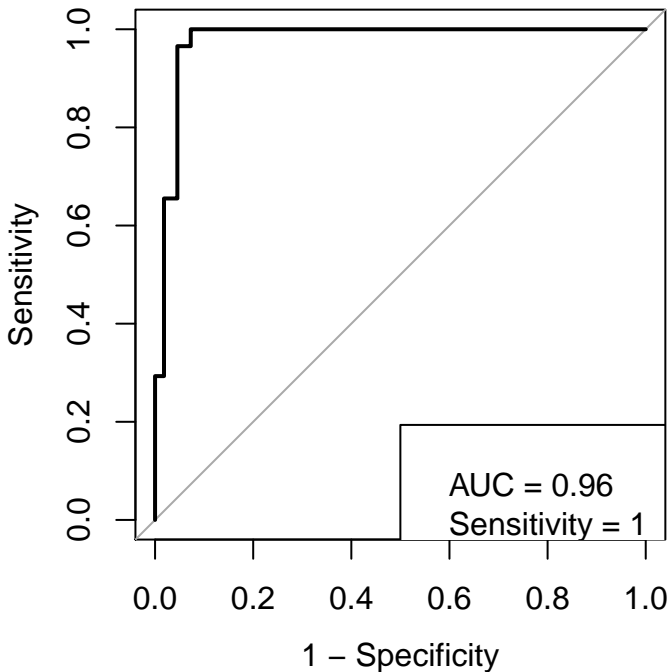

Supplement: Supplementary file 9 — Supplementary Software 1 [file 41467_2020_16141_MOESM9_ESM.zip › compute_code/output/Supp_Fig_5/ROC_olap.pdf]

# Estimated variant-specific effects and classification; olaparib

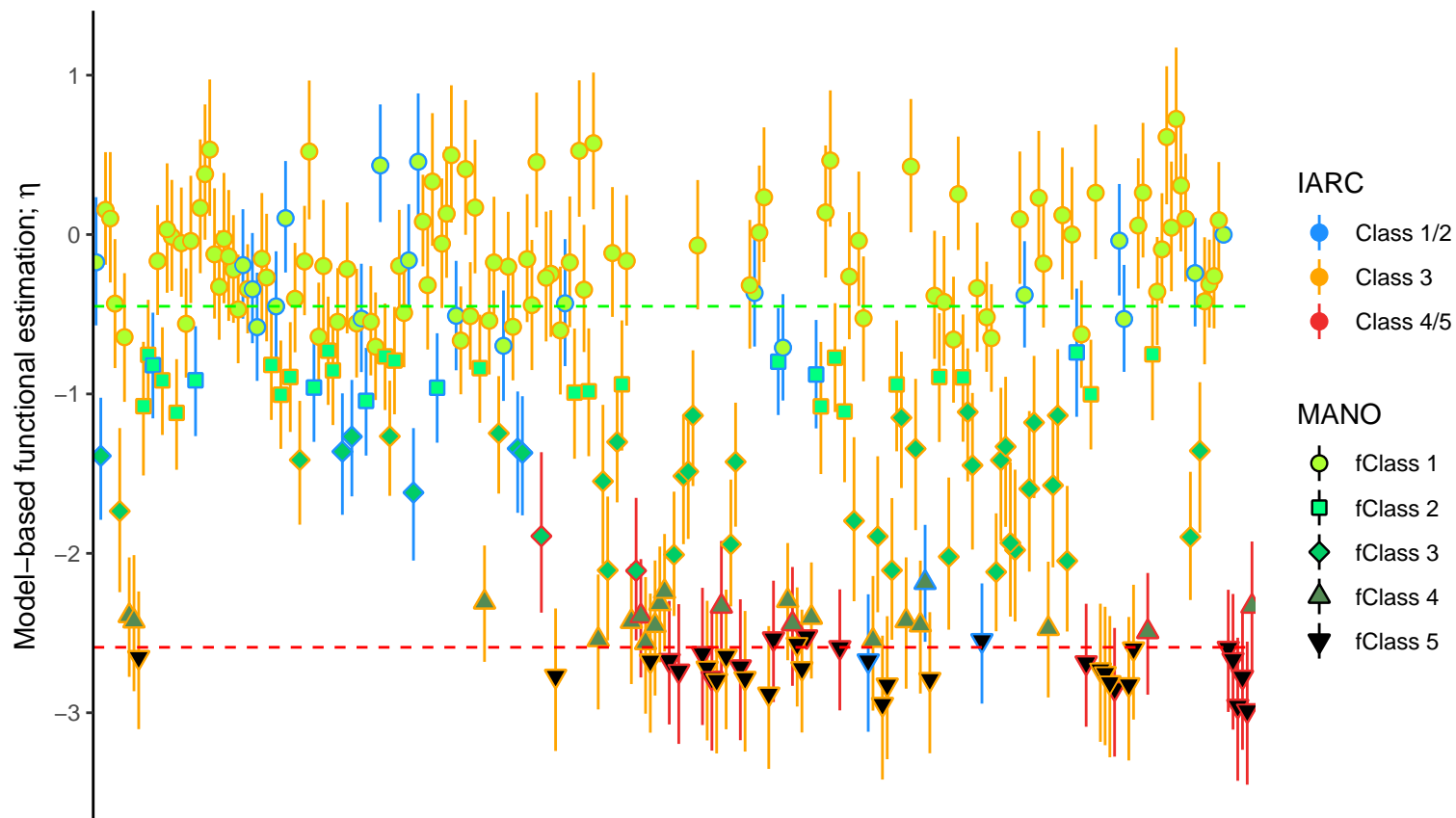

Supplement: Supplementary file 9 — Supplementary Software 1 [file 41467_2020_16141_MOESM9_ESM.zip › compute_code/output/Fig_3/Full-olaparib.pdf]

# Posterior predictive distribution curve and observed data

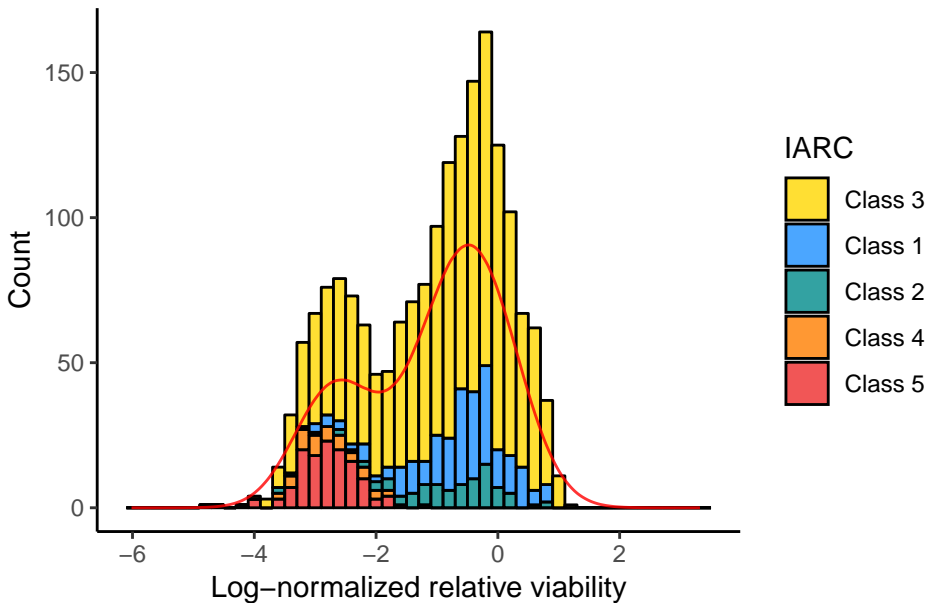

Supplement: Supplementary file 9 — Supplementary Software 1 [file 41467_2020_16141_MOESM9_ESM.zip › compute_code/output/Supp_Fig_10/Full-check-niraparib.pdf]

# Posterior predictive distribution curve and observed data

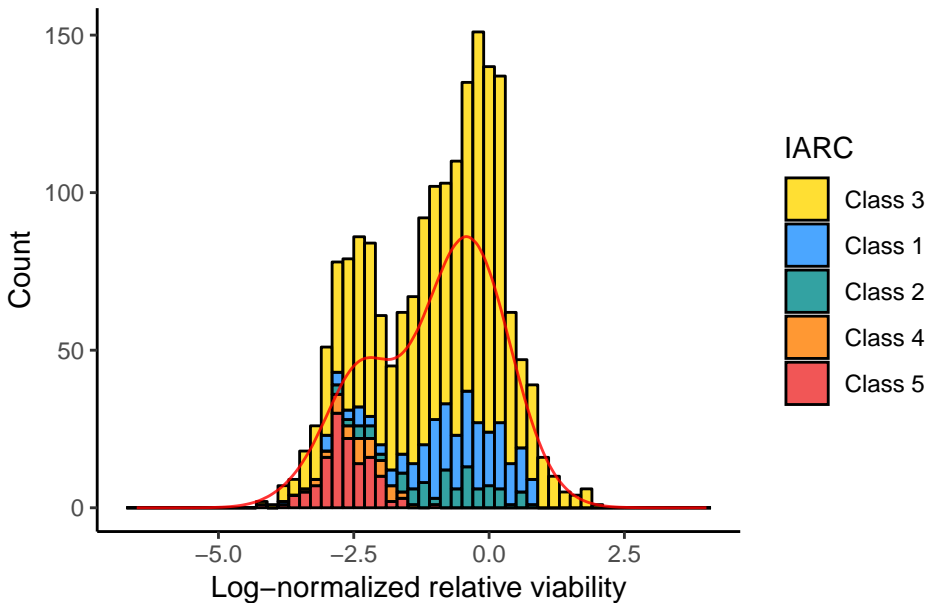

Supplement: Supplementary file 9 — Supplementary Software 1 [file 41467_2020_16141_MOESM9_ESM.zip › compute_code/output/Supp_Fig_10/Full-check-olaparib.pdf]

# Posterior predictive distribution curve and observed data

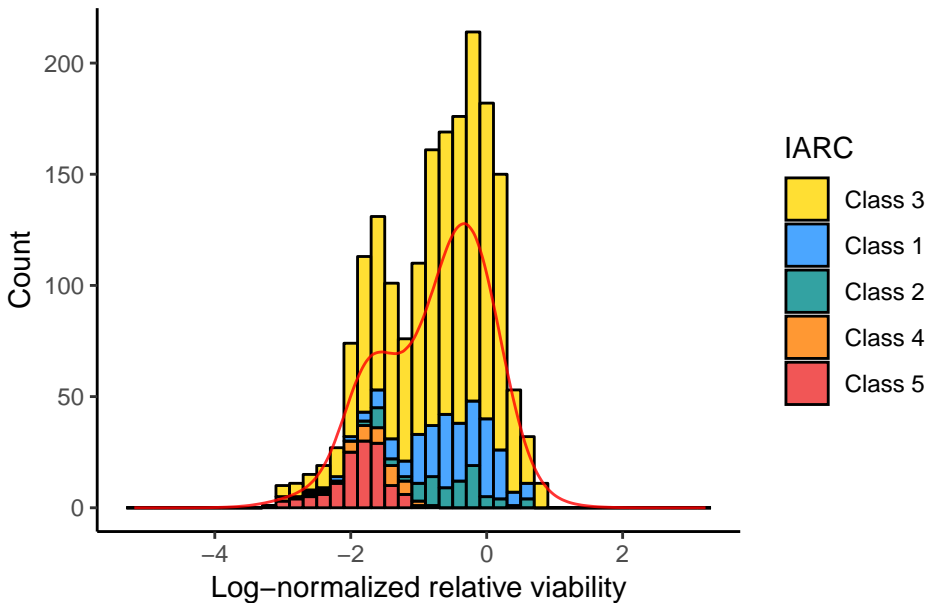

Supplement: Supplementary file 9 — Supplementary Software 1 [file 41467_2020_16141_MOESM9_ESM.zip › compute_code/output/Supp_Fig_10/Full-check-rucaparib.pdf]

Posterior predictive distribution curve and observed data

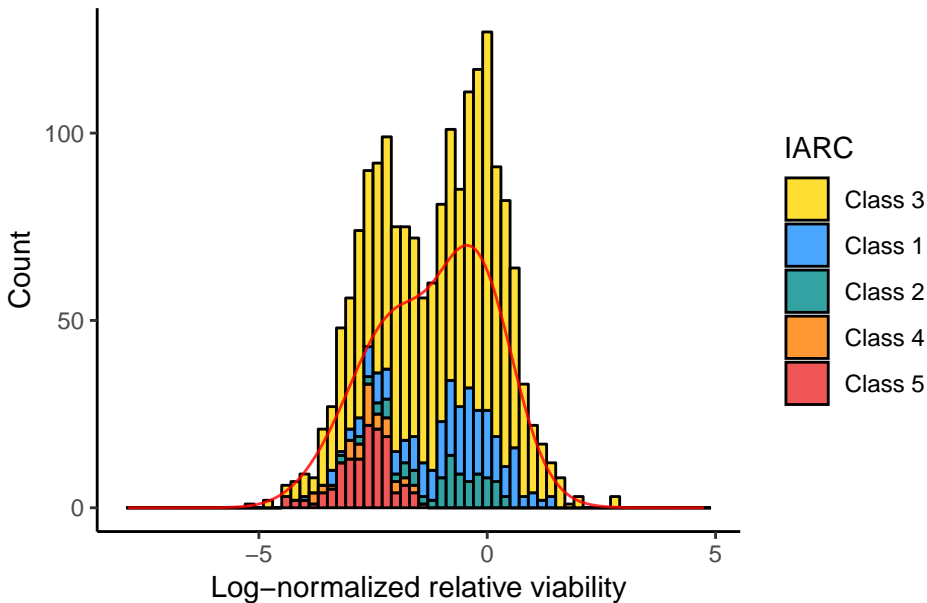

Supplement: Supplementary file 9 — Supplementary Software 1 [file 41467_2020_16141_MOESM9_ESM.zip › compute_code/output/Supp_Fig_10/Full-check-CBDCA.pdf]

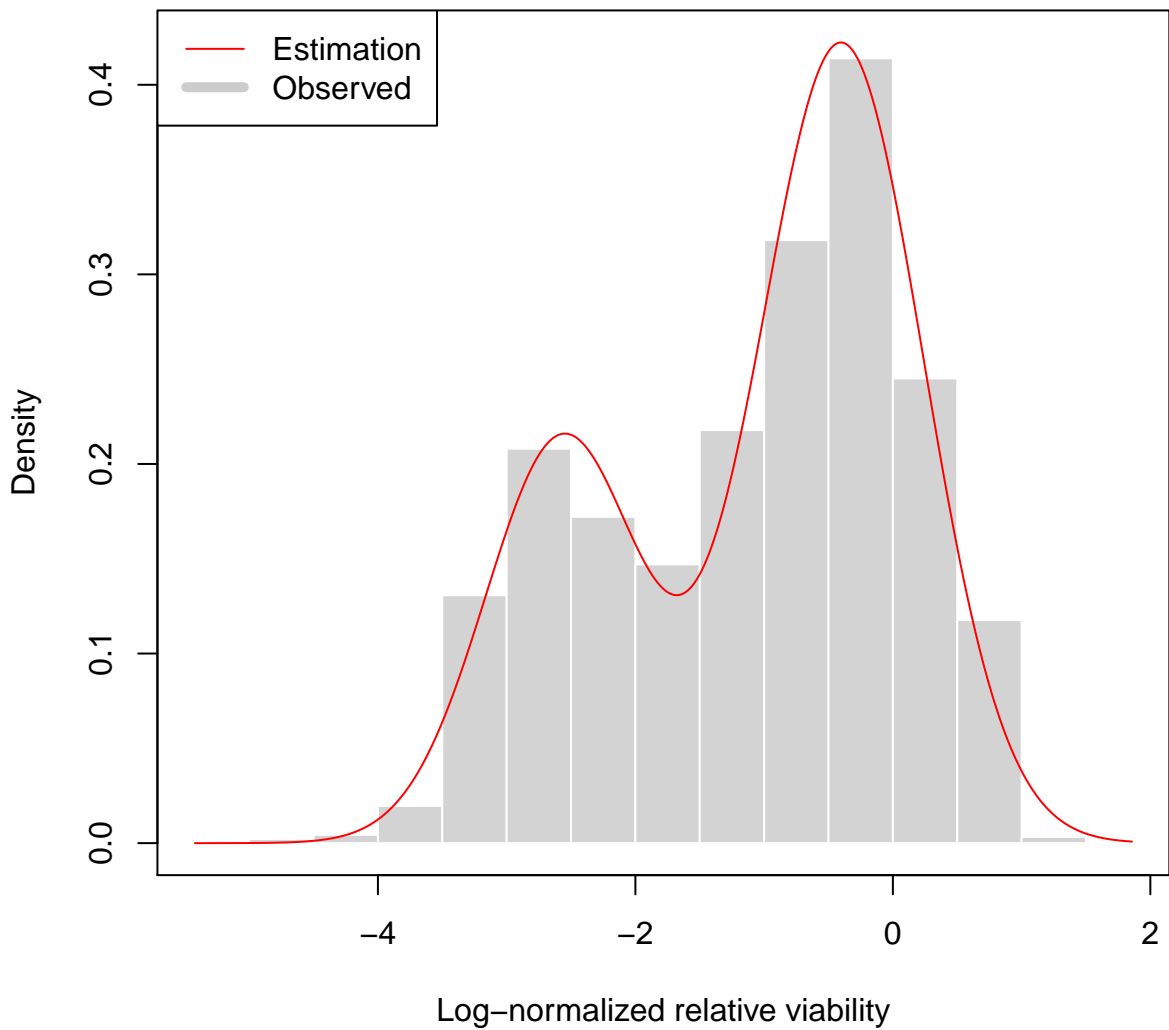

Supplement: Supplementary file 9 — Supplementary Software 1 [file 41467_2020_16141_MOESM9_ESM.zip › compute_code/output/Supp_Fig_6/distr_nira.pdf]

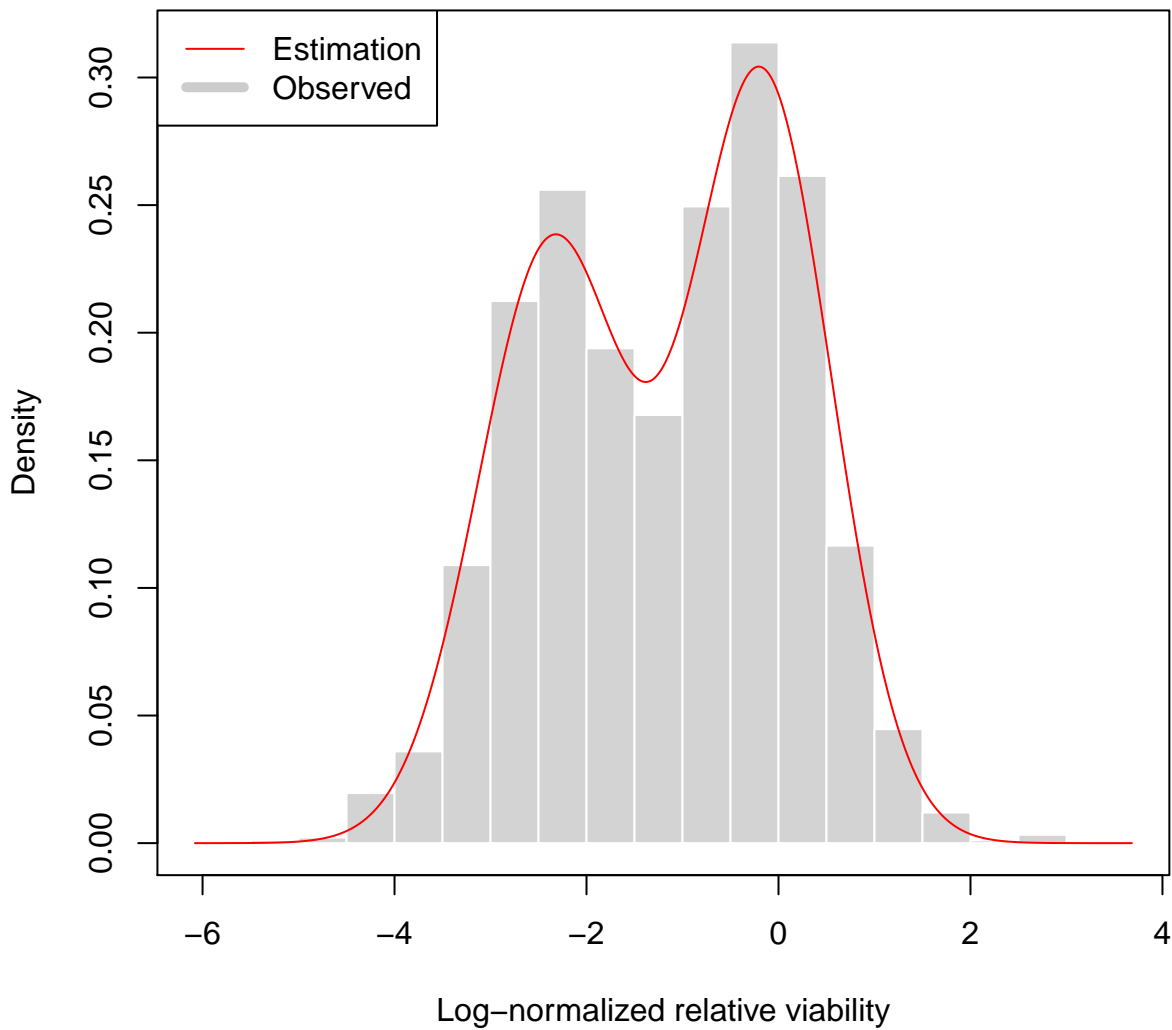

Supplement: Supplementary file 9 — Supplementary Software 1 [file 41467_2020_16141_MOESM9_ESM.zip › compute_code/output/Supp_Fig_6/distr_CDBCA.pdf]

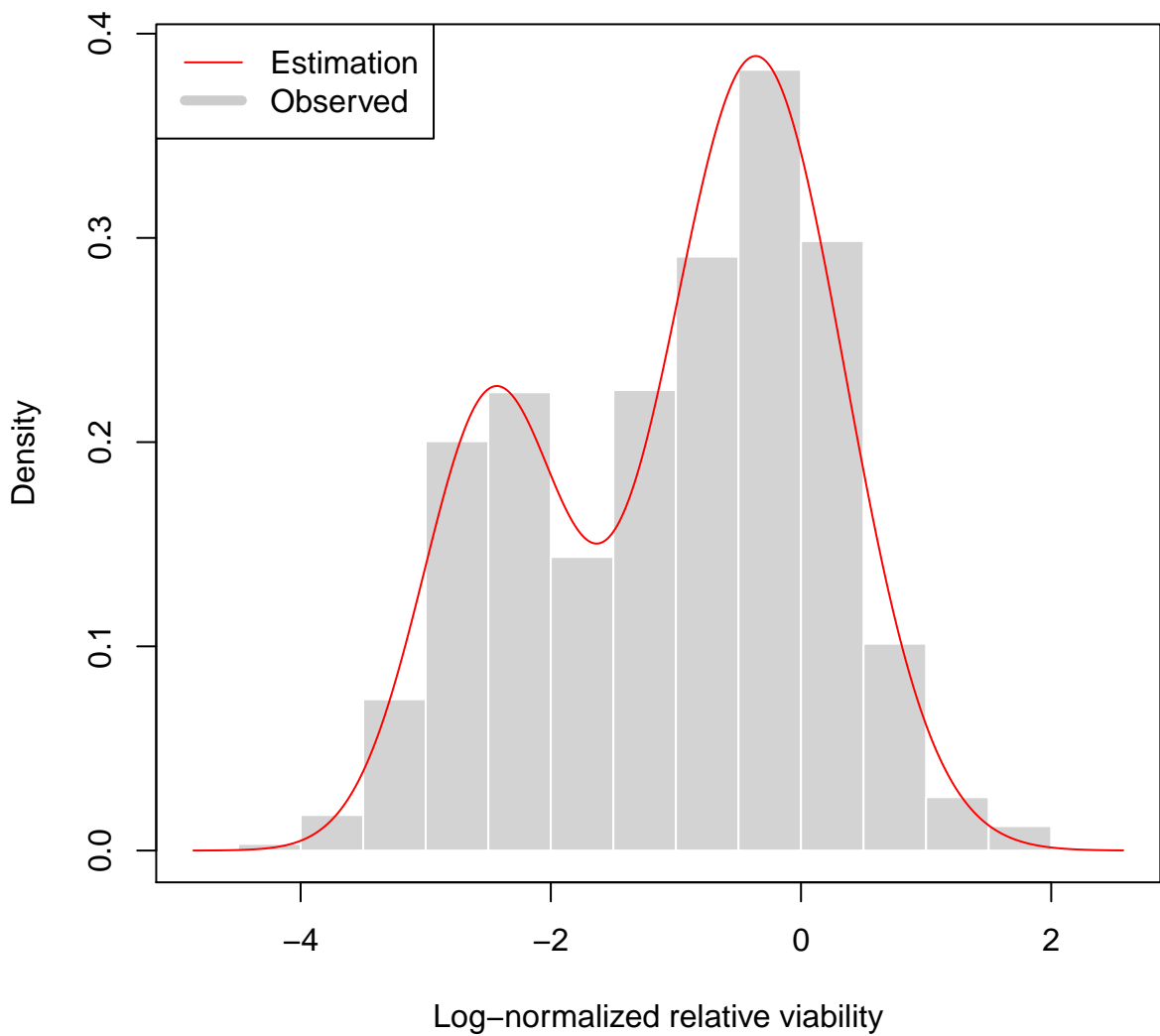

Supplement: Supplementary file 9 — Supplementary Software 1 [file 41467_2020_16141_MOESM9_ESM.zip › compute_code/output/Supp_Fig_6/distr_olap.pdf]

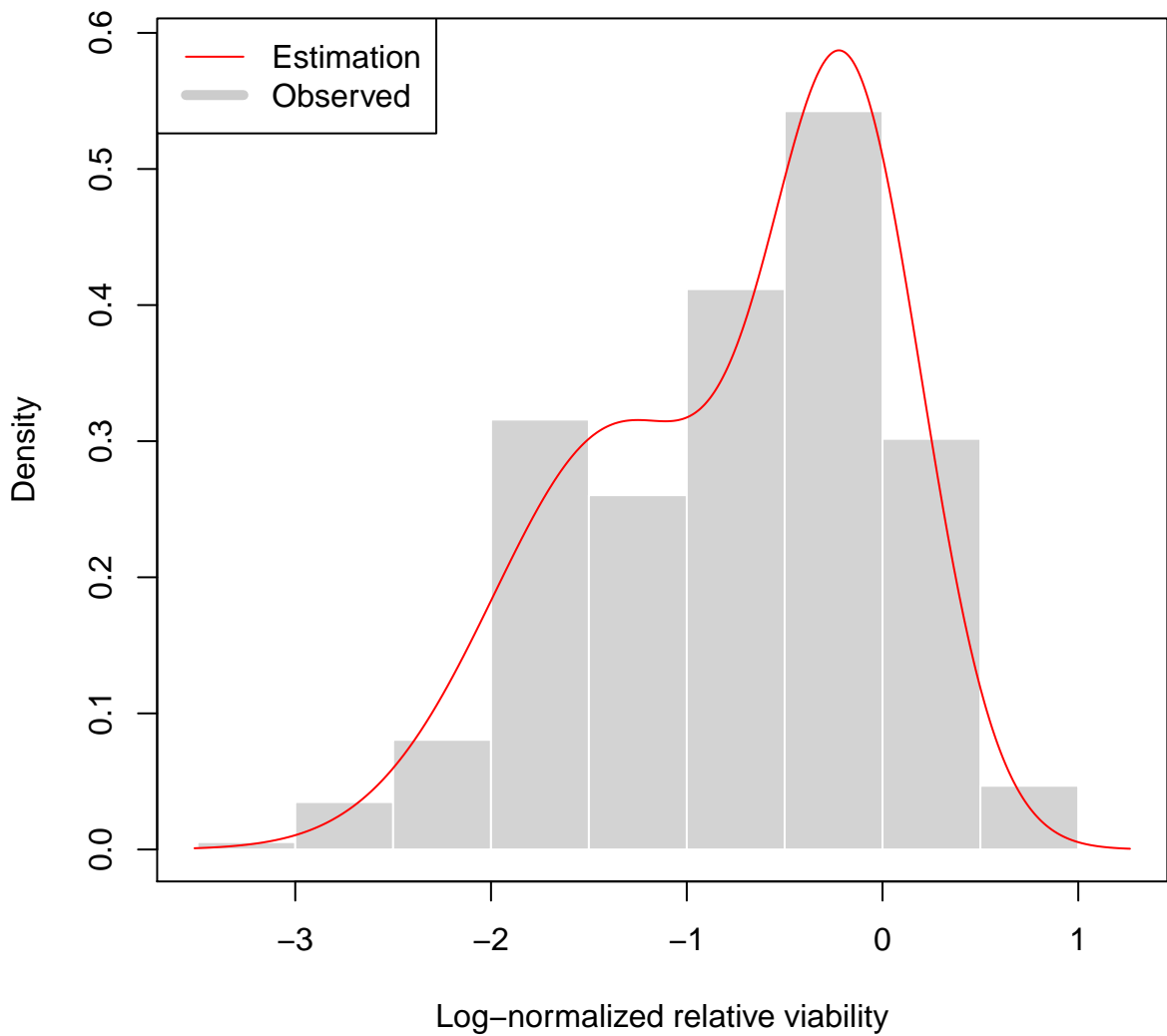

Supplement: Supplementary file 9 — Supplementary Software 1 [file 41467_2020_16141_MOESM9_ESM.zip › compute_code/output/Supp_Fig_6/distr_ruca.pdf]

# Estimated variant-specific effects and classification; niraparib

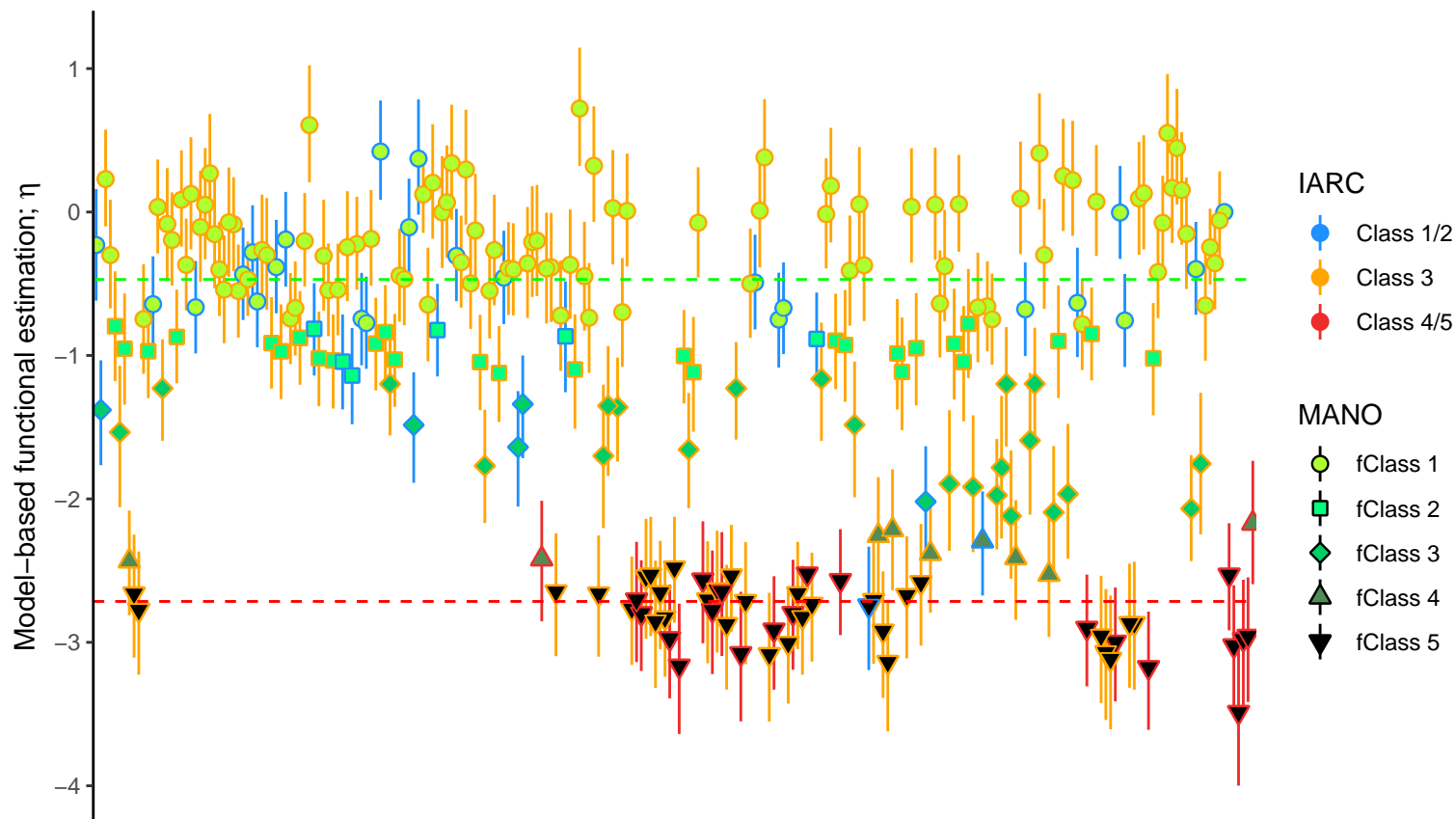

Supplement: Supplementary file 9 — Supplementary Software 1 [file 41467_2020_16141_MOESM9_ESM.zip › compute_code/output/Supp_Fig_9/Full-GVGD-niraparib.pdf]
