# Supplementary material for: High-throughput functional evaluation of BRCA2 variants of unknown significance
Source: Nat Commun. 2020 May 22;11:2573. doi: 10.1038/s41467-020-16141-8 (PMC7244490; doi:10.1038/s41467-020-16141-8)
Supplement: Supplementary file 10 — Reporting Summary [file 41467_2020_16141_MOESM10_ESM.pdf]

nature research

Corresponding author(s): Shinji Kohsaka
Hiroyuki Mano

Last updated by author(s): Mar 16, 2020

Reporting Summary

Nature Research wishes to improve the reproducibility of the work that we publish. This form provides structure for consistency and transparency in reporting. For further information on Nature Research policies, see Authors & Referees and the Editorial Policy Checklist .

Statistics

For all statistical analyses, confirm that the following items are present in the figure legend, table legend, main text, or Methods section.

n/a	Confirmed	
☐	☒	The exact sample size (*n*) for each experimental group/condition, given as a discrete number and unit of measurement
☐	☒	A statement on whether measurements were taken from distinct samples or whether the same sample was measured repeatedly
☐	☒	The statistical test(s) used AND whether they are one- or two-sided<br>*Only common tests should be described solely by name; describe more complex techniques in the Methods section.*
☐	☒	A description of all covariates tested
☐	☒	A description of any assumptions or corrections, such as tests of normality and adjustment for multiple comparisons
☐	☒	A full description of the statistical parameters including central tendency (e.g. means) or other basic estimates (e.g. regression coefficient) AND variation (e.g. standard deviation) or associated estimates of uncertainty (e.g. confidence intervals)
☐	☒	For null hypothesis testing, the test statistic (e.g. *F*, *t*, *r*) with confidence intervals, effect sizes, degrees of freedom and *P* value noted<br>*Give P values as exact values whenever suitable.*
☐	☒	For Bayesian analysis, information on the choice of priors and Markov chain Monte Carlo settings
☐	☒	For hierarchical and complex designs, identification of the appropriate level for tests and full reporting of outcomes
☒	☐	Estimates of effect sizes (e.g. Cohen's *d*, Pearson's *r*), indicating how they were calculated

Our web collection on statistics for biologists contains articles on many of the points above.

Software and code

Policy information about availability of computer code

Data collection	QuantaSoft (v.1.6.6.0320), BD FACSDiva Software (v6.1.3), PerkinElmer 2030 Software (v4.0), MiSeq Control Software (v2.5.0.5),
Data analysis	Bowtie2 v2.3.4.2, samtools v1.9, bcftools v1.9, and Integrative Genomics Viewer (IGV) v2.4.10 for sequencing BRCA2 variants.<br>SPiCE (v2.1.3) for assessing variant spliceogenicity.<br>R software v3.5.3 for statistical abalyses.<br>Stan language v2.19.3 for Bayesian inference.<br>mclust package v5.4.5 for expectation–maximization algorithm.<br>car package v3.0.6 for generating QQ plots.<br>GraphPad Prism software for Mac v8.02 for visualize the cell viability assay.<br>We used a custom made software running on R and Stan languages deposited at: https://github.com/MANO-B/Bayes

For manuscripts utilizing custom algorithms or software that are central to the research but not yet described in published literature, software must be made available to editors/reviewers. We strongly encourage code deposition in a community repository (e.g. GitHub). See the Nature Research guidelines for submitting code & software for further information.

Data

Policy information about availability of data

All manuscripts must include a data availability statement. This statement should provide the following information, where applicable:

- Accession codes, unique identifiers, or web links for publicly available datasets
- A list of figures that have associated raw data
- A description of any restrictions on data availability

All the raw data that support the findings of this study have been deposited in https://github.com/MANO-B/Bayes.

Field-specific reporting

Please select the one below that is the best fit for your research. If you are not sure, read the appropriate sections before making your selection.

☒ Life sciences ☐ Behavioural & social sciences ☐ Ecological, evolutionary & environmental sciences

For a reference copy of the document with all sections, see nature.com/documents/nr-reporting-summary-flat.pdf

Life sciences study design

All studies must disclose on these points even when the disclosure is negative.

Sample size

No sample size calculation was performed.
For digital droplet PCR, real-time qPCR, and cell viability assay, 20 BRCA2 variants and empty vector were used, so that each group contains at least five variants (six benign, five pathogenic, and ten unclassified variants).
For HDR assay, four additional variants (24 variants and empty vector in total) were utilized.
For MANO-B method, all the available BRCA2 variants at the time of each experiment (107 variants for the first examination, and 244 variants for the expanded examination) were utilized.

Data exclusions

No data exclusions were performed.

Replication

For MANO-B method, three biologically independent experiments were performed. Four drugs (olaparib, niraparib, rucaparib, and carboplatin) used in cell viability assay and MANO-B method showed similar results.
For real-time qPCR, three biological replicates were performed, and each with three technical replicates.
All the repeated experiments showed similar results. The number of replication for each experiment was described in figure legends.
For digital droplet PCR, a single experiment without replicates was performed.
For cell viability assay, five technical replicates for each variant were performed.
Reproducibility of cell viability assay was confirmed by two independent experiments.
Reproducibility of western blotting was confirmed by two independent experiments.
Bayesian inference was performed with four chains and the convergence was checked.

Randomization

Randomization was not performed for this study, because cells were randomly allocated to the various conditions.

Blinding

Blinding in this study was not performed.
All in vitro experiments whose data were generated by machine reading, blinding or not won't affect the results.
Data analysis were performed by the computational pipeline.

Reporting for specific materials, systems and methods

We require information from authors about some types of materials, experimental systems and methods used in many studies. Here, indicate whether each material, system or method listed is relevant to your study. If you are not sure if a list item applies to your research, read the appropriate section before selecting a response.

Materials & experimental systems

n/a	Involved in the study	
☐	☒	Antibodies
☐	☒	Eukaryotic cell lines
☒	☐	Palaeontology
☒	☐	Animals and other organisms
☒	☐	Human research participants
☒	☐	Clinical data

Methods

n/a	Involved in the study	
☒	☐	ChIP-seq
☐	☒	Flow cytometry
☒	☐	MRI-based neuroimaging

Antibodies

Antibodies used

anti-FLAG antibody (F3165; Sigma). anti-beta-Actin antibody (#4970; Cell Signaling Technology). peroxidase-linked secondary antibody for FLAG-BRCA2 (NA931V; GE Healthcare). peroxidase-linked secondary antibody for beta-Actin (NA934V; GE Healthcare)

Validation

All antibodies used in western blotting are obtained from commercial sources. Please see manufacturer's link for validation of antibody:

anti-FLAG antibody (F3165; Sigma)
https://www.sigmaaldrich.com/catalog/product/sigma/f3165

anti-beta-Actin antibody (#4970; Cell Signaling Technology)
https://en.cellsignal.jp/products/primary-antibodies/b-actin-13e5-rabbit-mab/4970

peroxidase-linked secondary antibody for FLAG-BRCA2 (NA931V; GE Healthcare) and peroxidase-linked secondary antibody for beta-Actin (NA934V; GE Healthcare)
https://www.gelifesciences.com/en/us/shop/protein-analysis/blotting-and-detection/blotting-standards-and-reagents/amersham-ecl-hrp-conjugated-antibodies-p-06260

Eukaryotic cell lines

Policy information about cell lines

Cell line source(s)	DLD1 parental cells and DLD1 BRCA2 (–/–) cells were purchased from Horizon Discovery.
Authentication	None of cell lines were authenticated by the authors.<br>The supplier authenticated the cell lines by STR profiling. Please see manufacturer's link for validation:<br>https://horizondiscovery.com/en/products/gene-editing/cell-line-models/PIFs/Human-DIP-Cell-Lines?nodeid=entrezgene-675
Mycoplasma contamination	None of cell lines were tested for mycoplasma contamination.
Commonly misidentified lines (See ICLAC register)	None of the cell lines used are listed in the ICLAC database.

Flow Cytometry

Plots

Confirm that:

- [x] The axis labels state the marker and fluorochrome used (e.g. CD4-FITC).
- [x] The axis scales are clearly visible. Include numbers along axes only for bottom left plot of group (a 'group' is an analysis of identical markers).
- [x] All plots are contour plots with outliers or pseudocolor plots.
- [x] A numerical value for number of cells or percentage (with statistics) is provided.

Methodology

Sample preparation	Freshly collected DLD1 cells filtered with a 35-µm cell strainer were used.
Instrument	BD FACSCanto II
Software	BD FACSDiva Software v6.1.3
Cell population abundance	After the preliminary FSC/SSC gating, 25,000–50,000 cells were analyzed for each sample.
Gating strategy	The preliminary FSC/SSC gating was applied for removing debris. GFP positive and negative populations were defined and gated by the negative control and the BRCA2 wild-type positive control.

- [x] Tick this box to confirm that a figure exemplifying the gating strategy is provided in the Supplementary Information.
